# Lung metastasis from thyroid cancer: A case report of unusual imaging presentation of lung metastases

**DOI:** 10.1097/MD.0000000000034733

**Published:** 2023-08-11

**Authors:** Wenjing Song, Shiwei Liu, Yuan Yu, Qian Xu, Shuzhen Liu, Jun Chen

**Affiliations:** a Medical Oncology, Dalian Medical University, Dalian, Liaoning, China; b Oncology Department, the First Affiliated Hospital of Weifang Medical University, Weifang, Shandong, China; c Joint surgery Department, the First Affiliated Hospital of Weifang Medical University, Weifang, Shandong, China; d Medical Oncology, the Second Hospital of Dalian Medical University, Dalian, Liaoning, China.

**Keywords:** case report, computed tomography, hemangiomas, lung metastasis, miliary tuberculosis, thyroid cancer, vascular malformations

## Abstract

Thyroid cancer (TC) is the most common malignancy of the head and neck and endocrine system. Distant metastases from TC are rare and are diagnosed in only 1% to 4% of patients, and these patients have a poor prognosis, which is the leading cause of TC-related deaths. There are few reports on metastatic TC in China and abroad, and even fewer reports on lung metastases from TC. We report a special patient with lung metastases of TC.

**Patient concerns:**

The patient is a 31-year-old female who was found to have both lung nodules during physical examination. Chest computed tomography (CT) showed that the density of both lung nodules was the same as the vascular density, considering that the possibility of vascular origin was not excluded.

**Diagnosis:**

After consultation with the whole hospital, it was considered that vascular malformations, hemangiomas, and malignant metastases were not excluded, the patient percutaneous lung biopsy had a high risk of bleeding, and thoracoscopic lobectomy could be performed in thoracic surgery to further clarify the pathology and diagnosis.

**Outcomes:**

The patient underwent thoracoscopic left lower lobe wedge resection on February 24, 2021. Postoperative pathology: (left lower lung mass) metastatic carcinoma, combined with morphology and immunohistochemistry, leaning toward thyroid follicular carcinoma lung metastasis. On May 27, 2021, the patient underwent “total thyroidectomy + lymph node dissection in the right cervical VI region.” Pathological examination: (right lobe and isthmus of the thyroid gland) papillary TC, follicular subtype, and classic type, with interstitial fibrosis. The patient was diagnosed with lung metastasis of TC.

**Lessons:**

This patient had the same CT value of lung metastases as the vascular CT value, which is relatively rare in our clinical practice and worthy of our study. The special CT imaging presentation of this TC patient with lung metastases further broadened our horizon. In clinical practice, when we encounter similar cases, we should combine more with other tests and examinations of patients to avoid misdiagnosis and missed diagnosis.

## 1. Introduction

Thyroid cancer (TC) is the most common malignancy of the head and neck and endocrine system, but has a relatively good prognosis with an incidence of 3.1% and a mortality rate of 0.7%.^[1]^ Distant metastases from TC are rare and are diagnosed in only 1% to 4% of patients. However, the prognosis of these patients is poor, which is the leading cause of TC-related deaths.^[2]^ TC metastasis sites are mainly distributed in the lungs, bones, brain, and liver, with the most common metastatic sites being the lungs (43%), followed by the bone (33%).^[3–7]^ Long-term overall survival for patients with lung metastases ranges from 25% to 75%.^[8,9]^ Considering the potential effect of all available treatment strategies, accurate assessment at diagnosis is a key factor in clinical decision making regarding the timing and type of initial treatment, including local or systemic therapy. The detection rate of TC has been increasing annually in recent years owing to the popularity of thyroid ultrasound for physical examinations. However, clinically metastatic TC is extremely rare, with only a few scattered clinical cases reported in China and other countries. In this article, we present a patient with pulmonary metastasis of TC, whose special point is the special imaging manifestation of pulmonary metastases, and hope to provide some reference and reflection to general medical practitioners.

## 2. Case presentation

**Figure 1.**
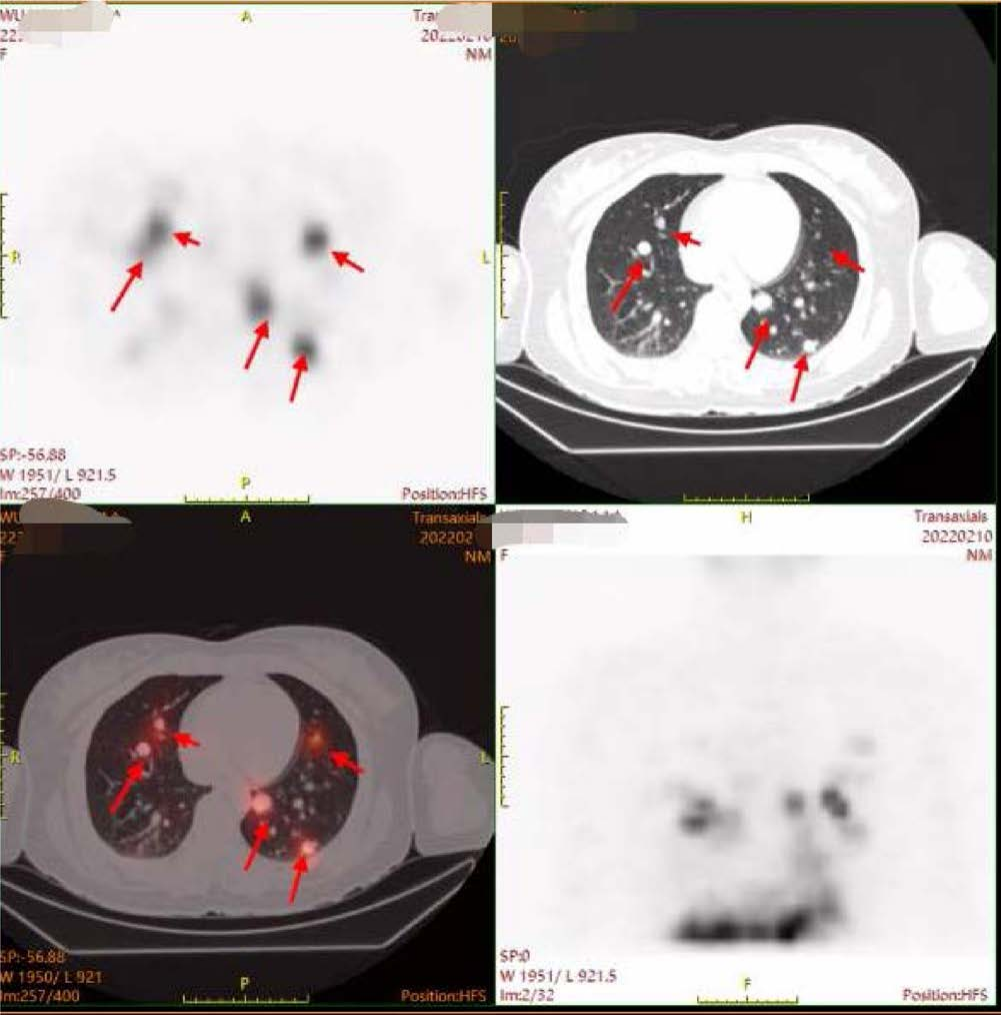
PET/CT of lung metastases in this patient. PET/CT = positron emission computed tomography.

**Figure 2.**
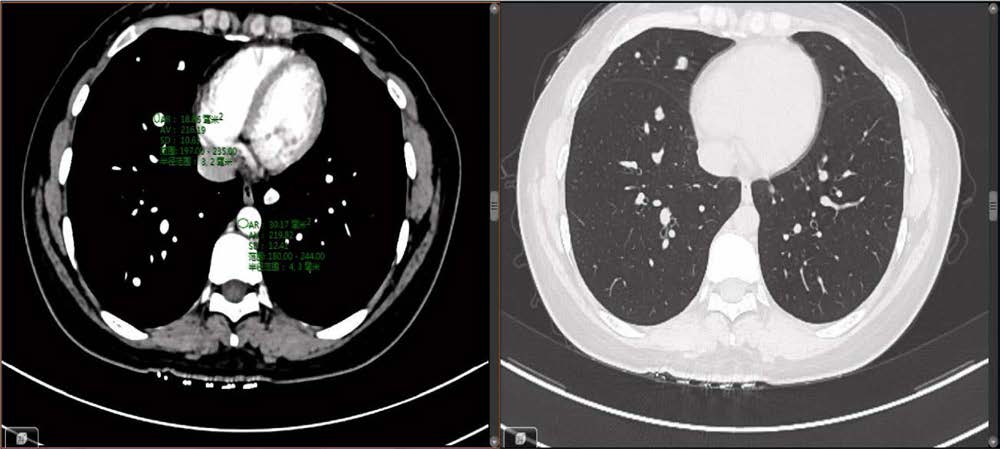
The CT presentation of lung metastases in this patient showed the same CT values as the vascular CT values. CT = chest computed tomography.

Patient, female, 31 years old, was admitted to our hospital with “bilateral lung nodules found for half a month.” Chest computed tomography (CT) revealed multiple nodules in both lungs during a checkup on 2021.02.03. Positron emission computed tomography (PET/CT) performed in our hospital on 2021.02.05 showed multiple solid nodules in both lungs (the largest was 1.4 × 1.3 cm, standard uptake value [SUV] max = 6.0), some nodules with slightly high metabolism (SUVmax = 0.9–6.0), not excluding metastases; fluid density shadow in the uterine cavity, no metabolism abnormalities were seen, mostly physiological changes; no significant metabolic abnormalities were seen in the remaining sites. As shown in Figure 1. Previous physical fitness, no history of special diseases such as tuberculosis, denial of family history of hereditary and similar diseases, no addiction to smoking, no addiction to alcohol, and no addiction to drugs. Unmarried, 1 pregnancy, 0 births, 1 miscarriage, last menstruation 2021.01.26. Physical examination revealed no enlargement of superficial lymph nodes. Both lungs were clear on percussion and breath sounds were clear on auscultation. Abdominal examination did not reveal any abnormalities. Supplementary investigations: 2021.02.03 Chest CT: multiple nodular shadows in both lungs, metastases to be ruled out. 2021.02.03 Breast ultrasound: bilateral breast hyperplasia. Relevant examinations were completed after admission to our hospital: tumor markers, neuron-specific enolase 18.77 ng/mL, residual tumor markers carbohydrate antigen 724, carbohydrate antigen 125, carbohydrate antigen 199, non-small cell lung cancer associated antigen, carcinoembryonic antigen, alpha-fetoprotein, squamous cell carcinoma-associated antigen, serum gastrin-releasing peptide precursor, tuberculosis infection T-cell spot test, TORCH, quantification of Aspergillus antigen, fungal D-glucan determination, procalcitonin, C-reactive protein, interleukin, and blood results were approximately normal. 2021.02.15 Thyroid ultrasound: multiple cystic and cystic-solid thyroid nodules, ACRTI-RADS category 3; solid nodules in the right lobe of the thyroid (size 0.3 × 0.3 cm), ACRTI-RADS category 4. 2021.02.05 Transvaginal uterine adnexal ultrasound: A small amount of fluid in the uterine cavity. 2021.02.07 Cervical exfoliative cytology test revealed no intraepithelial lesions or malignant lesions, and moderate inflammation. 2021.02.16 Cervicothoracic abdominal pelvic CT: hypodense foci in the left lobe of the thyroid; multiple nodules in both lungs, lesions of vascular origin? Further clinical examination is indicated to exclude rich blood supply metastases; uterine changes, and pelvic effusions. As shown in Figure 2. 2022.02.17 Cranial magnetic resonance imaging revealed no significant abnormalities. Relevant examinations were performed after the patient was admitted to our hospital. After discussion, the general physicians concluded that the patient had multiple nodules in both lungs, and the possibility of metastases was high in combination with relevant tests and examinations. The patient previous breast and gynecological ultrasounds did not show any significant abnormalities. CT showed that the density of both lung nodules was the same as the density of blood vessels, and the possibility of a vascular origin was not excluded. The risk of percutaneous lung puncture bleeding was high, and pulmonary biopsy was not considered for the time being to clarify the pathology. The whole hospital consultation was requested to help clarify the condition and further diagnosis and treatment. Thyroid ultrasound showed a nodule in the right lobe of the thyroid gland with ACRTI-RADS category 4, which was considered malignant, and a puncture biopsy could be performed for further clarification.

Consultation opinion of the imaging department of our hospital: After a comprehensive review of the radiographs, the possibility of vascular malformation, congenital aneurysm, hemangioma, etc, could be considered according to the imaging results, and further pathology could be clarified if a definite diagnosis is needed. Nuclear medicine physician consultation opinion: Combined with the patient PET/CT results, the patient double lung multiple nodules, the edge is smoother, the density is more uniform, SUVmax = 0.9 to 6, the largest is located in the lower lobe of the left lung, the size is 1.4 × 1.3 cm, SUVmax = 6.0, the metabolic value of the patient double lung nodules is higher, considering the possibility of mass proliferation, to exclude the possibility of sarcoidosis, not to exclude the possibility of malignancy, it is recommended to further clarify the pathology. Radiotherapist consultation opinion: The patient CT and PET/CT showed that multiple nodules in both lungs are likely to be malignant, but benign lesions are not excluded, CT shows that some nodules have vascular supply, considering the possibility of vascular variation, it is recommended to further clarify the pathology. Respiratory physician consultation opinion: The patient occupation was office clerk and could exclude asbestosis and other occupational diseases. Bronchoscopic lavage is feasible for further lavage fluid exfoliation cytology pathology, but the positive rate of this test is low, and gastrointestinal examinations can be further improved to exclude gastrointestinal lesions. Thoracic surgeon consultation opinion: Combined with the patient relevant tests and examination results, consider not excluding the possibility of vascular variation, aneurysm, and also not excluding the possibility of malignancy. Computed tomography (CT) of the lung nodules is the same as the vascular CT value, considering that the bleeding of percutaneous lung biopsy may be large. There are no other noninvasive auxiliary tests that can further help clarify the diagnosis, and the remaining auxiliary examinations do not show obvious contraindications to surgery, with surgery to take pathological biopsy indications. Thoracoscopic lobe wedge resection can be performed, and pathological examination can be performed after surgery to clarify the condition. The summary opinion of the whole hospital consultation is as follows: The patient currently has multiple nodules in both lungs, considering the possibility of vascular malformation, hemangioma, and malignant metastases; the patient is currently at high risk of bleeding from percutaneous pulmonary puncture; he can undergo thoracoscopic lobectomy in thoracic surgery to further clarify the pathology and diagnosis, and can also be reexamined after 2–3 months to clarify the changes in the mass to determine further treatment. The patient and his family were informed of consultation and further treatment. After discussion, the patient and his family expressed their request to clarify the pathology in the thoracic surgery department, and then transferred to the thoracic surgery department of our hospital for the clarification of diagnosis and further treatment.

**Figure 3.**
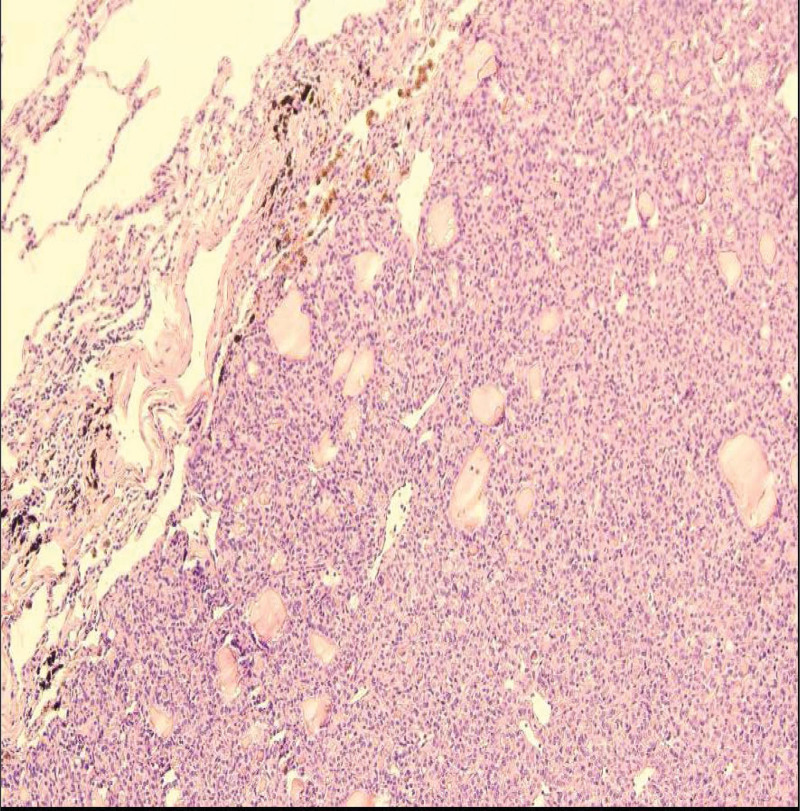
Pathology after wedge resection of the lower lobe of the left lung.

On February 20, 2021, the patient was transferred to our thoracic surgery department, and on February 22, 2021, electronic bronchoscopy was performed without any significant abnormality. The preoperative discussion concluded that the mass of the outer basal segment of the lower lobe of the left lung was easier to resect, the trauma and surgical scope were minimal, and the proposed surgical plan was “thoracoscopic wedge resection of the left lower lobe of the lung,” and the specific surgical resection range was determined by rapid pathology and intraoperative exploration. Thoracoscopic wedge resection of the lower lobe of the left lung was performed on February 24, 2021. Endoscopic examination showed no obvious adhesion in the chest cavity, no effusion, pulmonary fissure development, multiple masses in both lungs, hemangiomatous bump-like changes on the pleural surface, tenacity, and no obvious enlarged lymph nodes under the hilar and carina, and the operation was successful. 2021.03.31 Postoperative pathology: (left lower lobe lung mass) metastatic carcinoma, combined with morphology and immunohistochemistry, leaning toward pulmonary metastasis of thyroid follicular carcinoma, with 3 masses, volume 1.5 cm × 1.4 cm × 1 cm, 1.2 cm × 1.2 cm × 0.7 cm and 0.4 cm in diameter, involving the pulmonary membrane, without involvement of fine bronchi, with net lung sections; no clear vascular cancer embolus and nerve invasion. As shown in Figure 3. Immunohistochemistry: CK wide (+), TTF-1 (+), CK7 (+), CK19 (weak +), CyclinD1 (+), CD56 (partial +), TG (+), NapsinA (−), PTH (−), Galectin-3 (−), Ki-67 index (5%). The patient recovered well after the surgery and was later given a discharged. The patient was finally diagnosed with lung metastasis of TC. The patient later underwent ultrasound-guided right-sided thyroid puncture biopsy, and cytology revealed a suspicious follicular tumor. On May 27, 2021, “total thyroidectomy + lymph node dissection of right cervical VI area” was performed in the Cancer Hospital of the Chinese Academy of Medical Sciences. Pathological examination (right lobe and isthmus of the thyroid gland) revealed papillary carcinoma, follicular subtype, and classic type, with interstitial fibrosis. The tumor was trifocal, 0.3 to 0.5 cm in diameter, and the larger one involved extraperitoneal fibrofatty tissue of the thyroid gland. No clear vascular cancer embolus or nerve invasion was observed. The surrounding thyroid gland showed a nodular goiter with hemiadenomatous hyperplasia. (Precricothyroid tissue) 0/3, with little additional thyroid tissue seen as nodular goiter. (left lobe of the thyroid gland) nodular goiter with focal hemiadenomatous hyperplasia and active follicular epithelial hyperplasia. No metastatic carcinomas were observed in the lymph nodes (0/7). (right cervical zone 6a lymph node) 0/1. (right cervical zone 6b lymph nodes) 0/3. pTNM stage: pT1a(m)N0. Subsequently, the patient underwent iodine-131 treatment in our hospital at 2021.08.10, 2022.02.08, and 2022.08.09, and the doses were 150 mCi, 200 mCi, and 200 mCi, respectively, and the patient had no obvious discomfort after treatment.

## 3. Discussion

The different biological characteristics of the major entities of TC, such as papillary, follicular, medullary, and poorly differentiated, depend largely on their different metastatic spreads.^[10]^ Papillary TC has a propensity for lymphatic spread in the neck, occurring in 20% to 50% of patients, while distant metastases occur in <5% of cases. Lymph node metastasis in the neck may be the first symptom, especially in (micro) papillary TC.^[10]^ In contrast, follicular TC has a clear tendency for vascular invasion, but not lymphatic invasion. Distant metastasis occurs in 10% to 20% of follicular TC.^[10]^ Distant metastasis is the main negative prognosis for TC. Pulmonary metastases are common in metastatic diseases of differentiated thyroid cancers (DTCs). Owing to its insidious onset and slow progression, clinical diagnosis is relatively difficult.^[11]^ Most metastatic lung tumors are round, solid, and have well-defined nodular shadows on images. Lung metastases from TC usually present as multiple small nodules or miliary patterns in both lungs, which should be differentiated from lung cancer and miliary tuberculosis.^[12]^ There are few reports on metastatic TC in China and abroad, and even fewer reports on lung metastases from TC. We report a special patient with lung metastases of TC, whose CT values of lung metastases were the same as those of vascular CT, which is relatively rare in our clinical practice and worthy of consideration. Previous studies have shown that advanced age, follicular carcinoma, poorly differentiated tumors, tumor size >10 mm, lymphatic metastasis, and extraperitoneal invasion are associated with lung metastasis of TC.^[13]^ The current clinical diagnosis of DTC patients with or without lung metastases is routinely made using I131 whole-body imaging, serum thyroglobulin (Tg), chest CT, PET/CT, etc. The performance of lung metastases in DTC patients on chest CT is nonspecific, so the combination of I131 whole-body imaging, Tg, PET/CT, and needle biopsy of lung metastases in patients with suspected lung metastases is the key to improve the diagnostic accuracy, and physicians should combine diagnostic protocols in clinical treatment to reduce the risk of misdiagnosis and omission, reduce medical disputes, and comprehensively improve the prognosis of patients.

## 4. Conclusions

The special CT imaging presentation of this TC patient with lung metastases further broadened our horizon. In clinical practice, when we encounter similar cases, we should combine more with other tests and examinations of patients to avoid misdiagnosis and missed diagnosis.

## Author contributions

**Conceptualization:** Yuan Yu.

**Data curation:** Wenjing Song.

**Methodology:** Shuzhen Liu.

**Supervision:** Yuan Yu, Shuzhen Liu, Jun Chen.

**Visualization:** Qian Xu.

**Writing – original draft:** Wenjing Song, Shiwei Liu.

**Writing – review & editing:** Jun Chen.
